# Photoactivatable oncolytic adenovirus for optogenetic cancer therapy

**DOI:** 10.1038/s41419-020-02782-6

**Published:** 2020-07-23

**Authors:** Yasuko Hagihara, Ayaka Sakamoto, Takashi Tokuda, Tomoki Yamashita, Sena Ikemoto, Ayaka Kimura, Makito Haruta, Kiyotaka Sasagawa, Jun Ohta, Kazuo Takayama, Hiroyuki Mizuguchi

**Affiliations:** 1 Laboratory of Biochemistry and Molecular Biology, Graduate School of Pharmaceutical Sciences, Osaka University, Osaka, 565-0871 Japan; 2 Laboratory of Hepatocyte Regulation, National Institutes of Biomedical Innovation, Health and Nutrition, Osaka, 567-0085 Japan; 3 Laboratory for Future Interdisciplinary Research of Science and Technology, Institute of Innovative Research, Tokyo Institute of Technology, Tokyo, 152-8550 Japan; 4 PRESTO, Japan Science and Technology Agency, Saitama, 332-0012 Japan; 5 Division of Materials Science, Graduate School of Science and Technology, Nara Institute of Science and Technology, Ikoma, 630-0192 Japan; 6 Global Center for Medical Engineering and Informatics, Osaka University, Osaka, 565-0871 Japan; 7 Integrated Frontier Research for Medical Science Division, Institute for Open and Transdisciplinary Research Initiatives (OTRI), Osaka University, Osaka, 565-0871 Japan

**Keywords:** Tumour virus infections, Tumour virus infections

## Abstract

Virotherapy using oncolytic adenovirus is an effective anticancer strategy. However, the tumor selectivity of oncolytic adenoviruses is not enough high. To develop oncolytic adenovirus with a low risk of off-tumor toxicity, we constructed a photoactivatable oncolytic adenovirus (paOAd). In response to blue light irradiation, the expression of adenoviral E1 genes, which are necessary for adenoviral replication, is induced and replication of this adenovirus occurs. In vitro, efficient lysis of various human cancer cell lines was observed by paOAd infection followed by blue light irradiation. Importantly, there was no off-tumor toxicity unless the cells were irradiated by blue light. In vivo, tumor growth in a subcutaneous tumor model and a mouse model of liver cancer was significantly inhibited by paOAd infection followed by blue light irradiation. In addition, paOAd also showed a therapeutic effect on cancer stem cells. These results suggest that paOAd is useful as a safe and therapeutically effective cancer therapy.

## Introduction

Virotherapy using oncolytic adenovirus is an effective anticancer strategy. Among various oncolytic virus, adenovirus (43%) is the most commonly used viral platform^1^. Oncolytic adenoviruses are human adenoviruses that have been genetically engineered in such a way as to replicate in tumor cells and destroy the tumor. The use of oncolytic adenoviruses as anticancer agents is eagerly anticipated, since these viruses exhibit multiple tumor-targeting mechanisms for enhanced antitumor properties. However, the tumor selectivity of oncolytic adenoviruses is not sufficiently high, and thus their safety must be improved. We considered that introduction of optogenetic techniques into oncolytic adenoviruses might achieve a major enhancement of tumor selectivity. Through the development of light-activated oncolytic adenoviruses, high tumor selectivity could be realized by simple optical illumination. Our objective in this study was thus to generate photoactivatable oncolytic adenoviruses (paOAd) that can be used as safe and effective anticancer agents.

Our group previously developed tumor-specific, replication-competent adenovirus oncolytic adenoviruses (TRADs) for cancer treatment^2,3^. Fujiwara and his colleagues constructed a TRAD, in which the human telomerase reverse transcriptase (hTERT) promoter element drives expression of the adenovirus early region 1A (E1A) and adenovirus early region 1B (E1B) genes, which are necessary for adenoviral replication^4^. TRAD induces selective E1A and E1B expression in hTERT-positive human cancer cells. However, some of the normal cells and tissues, such as somatic stem cells, and cells of the testis, bone marrow, and duodenum, are also positive for hTERT. Thus, TRADs run a risk of unexpected replication in normal cells.

## Materials and methods

### Plasmid construction

Ad vectors were prepared by an improved in vitro ligation method^2,3^. The hTERT-GAVPO-pA-UASG-E1A-2A-E1B19k-pA (hTERT promoter, GAVPO: VVD (the smallest light-oxygen-voltage (LOV) domain-containing protein), Gal4 (Gal4 residues 1–65), and p65 activation domain, pA: poly(A) signal, UAS: upstream activating sequence of Gal, E1A: adenovirus early region 1A, 2A: 2A self-cleaving peptides, E1B19k: adenovirus early region 1B 19 kDa protein) cassette was generated by using a Standard GENE SYNTHESIS service (GENEWIZ). pUC57-hTERT-GAVPO-pA-UASG-E1A-2A-E1B19k-pA was digested with I-CeuI/PI-SceI and then ligated with I-CeuI/PI-SceI-digested pAdHM3 (ref. ^2^), resulting in pAdHM3-hTERT-GAVPO-pA-UASG-E1A-2A-E1B19k-pA. Ad-hTERT-LacZ was similarly constructed. Further details on the plasmid construction are available upon request.

### Photoactivatable oncolytic adenovirus

The paOAd was prepared as follows. PacI-digested pAdHM3-hTERT-GAVPO-pA-UASG-E1A-2A-E1B19k-pA were transfected into HEK293 cells by using Lipofectamine 2000 (Thermo Fisher Scientific), resulting in AdHM3-hTERT-GAVPO-pA-UASG-E1A-2A-E1B19k-pA (named as paOAd). paOAd was amplified and purified by two rounds of cesium chloride gradient ultracentrifugation, dialyzed, and stored at −80 °C. As a control, conventional oncolytic adenoviruses were used as described in our previous study^5^. In conventional oncolytic adenovirus, E1A and E1B genes are controlled by hTERT promoter. As a result, this oncolytic adenovirus selectively replicates in hTERT-positive cells. These recombinant Ads were amplified and purified by two rounds of cesium chloride gradient ultracentrifugation, dialyzed, and stored at −80 °C (ref. ^5^). The infectious units (IFU) were calculated by using an Adeno-X Rapid Titer Kit (Clontech) according to the manufacturer’s instructions. The particle to infectivity ratio of paOAd was ~12–15: 1.

### In vitro illumination

The cells were illuminated by using an Ultra High Power LED (Prizmatix). Blue light was measured by using a Photodiode Power Sensor (PD300) held directly above the light-emitting diode (LED). The cells were illuminated from the bottom of the dish. To establish a dark condition, the cell-containing plates were wrapped in aluminum foil.

### In vivo illumination

The implantable blue LED devices were produced as follows. A polyimide flexible substrate with Cu wiring patterns was used as the base structure for the implantable blue LED devices. The substrate was manufactured using the standard flexible substrate technology used in the electronics industry. The thickness of the substrate was 110–150 µm. A commercially available InGaN/Sapphire blue LED with an emission peak wavelength of 470 nm was employed for the device. Three to five LEDs were attached to the end of the flexible substrate with a transparent epoxy resin, and then the LEDs were connected to the wiring patterns on the substrate. A standard wire-bonding technique for IC chips was used for the connection process. The LEDs were series connected to achieve a same current condition during operation. Then the ends of the implantable LED devices were molded with epoxy resin for waterproofness. Finally, after the epoxy resin had cured, a parylene layer with a thickness of 5–10 µm was deposited for better biocompatibility. LED devices were inserted between the right and left lobes of the mouse. By fixing the power cord to the peritoneum, the LED devices were prevented from moving outside the liver. LED devices were inserted in both the “paOAd + dark” and “paOAd + light” groups. Only the “paOAd” group was irradiated with blue light.

### Treatment of subcutaneous xenograft model mice

H1299 or HepG2 cells (3 × 10^6^ cells in 50 μL PBS) were mixed with 50 μL Matrigel (BD Biosciences) and injected subcutaneously into the flanks of 8-week-old female BALB/c *nu/nu* mice (Japan SLC Inc.). When the tumors had grown to ~5–6 mm in diameter, the mice were intratumorally injected with paOAd at a dose of 1 × 10^7^ IFU/mouse. At day 3 after the first injection, the mice were given an additional intratumoral injection of paOAd at a dose of 1 × 10^7^ IFU/mouse. At 24 h after the first infection, the tumor was irradiated with 90 mW/cm^2^ blue light for 10 days (8 h/day). The tumors were measured every week, and the tumor volume was calculated by the following formula: tumor volume (mm^3^) = *A* × *B*^2^ × 3.14 × 6^–1^, where *A* is the longest diameter and *B* is the shortest. These experiments were approved by the Animal Experiment Committee of the National Institutes of Biomedical Innovation, Health and Nutrition (NIBIOHN) and Osaka University.

### Liver cancer treatment model

HepG2 cells (1 × 10^6^ cells in 100 μL PBS) were injected intrasplenically into 10-week-old female *Rag2/Il2rg* double-knockout mice (Taconic Biosciences), were intraperitoneally infused with 0.5 ml/kg CCl_4_ for 4 weeks (twice a week) before the cell transplantation. In order to efficiently engraft HepG2 in the mouse liver, immunodeficient mice (*Rag2/Il2rg* double-knockout mice) in which liver injury was caused by CCl_4_ were used. Simultaneously with the transplantation of HepG2 cells, the implantable blue LED device was transplanted into the mouse liver. When the human alpha fetoprotein (AFP) secretion level in mouse serum reached 50 ng/ml, the mice were intravenously injected with paOAd at a dose of 5 × 10^9^ IFU/mouse. At day 3 after the first injection, the mice were given an additional intravenous injection of paOAd at a dose of 5 × 10^9^ IFU/mouse. At 24 h after the first infection, the liver was irradiated with 1 mW/cm^2^ blue light for 14 days (6 h/day). Because blue light does not readily penetrate tissue, only the livers were illuminated by blue light; other organs were not. In the subcutaneous xenograft model mice, strong blue light (90 mW/cm^2^) was used to irradiate light from outside the mouse body. On the other hand, slightly weak blue light (1 mW/cm^2^) was used in the liver cancer treatment model because LED devices were directly inserted into the mouse liver. These experiments were approved by the Animal Experiment Committee of the National Institutes of Biomedical Innovation, Health and Nutrition (NIBIOHN) and Osaka University.

### Cancer cell line culture methods

HepG2 and HEK293 cells were cultured with D-MEM (High Glucose) with L-glutamine and phenol red (FUJIFILM Wako) containing 10% fetal bovine serum and penicillin–streptomycin. H1299 cells were cultured with RPMI-1640 with L-glutamine and phenol red (FUJIFILM Wako) containing 10% fetal bovine serum and penicillin–streptomycin. A549 cells were cultured with minimum essential medium eagle (FUJIFILM Wako) containing MEM nonessential amino acids solution (Thermo Fisher Scientific), 10% fetal bovine serum, and penicillin–streptomycin.

### Normal cell culture methods

Human umbilical vein endothelial cells (HUVEC) were purchased from Lonza and cultured using an EGM-2 endothelial cell growth medium-2 BulletKit (Lonza). Human small intestinal organoids (SIO) were generated from human iPS cells using a STEMdiff intestinal organoid kit (STEMCELL Technologies). The method for culturing human iPS cells was performed according to our previous reports^6,7^.

### ELISA

Mouse serum samples were collected periodically from the orbital plexus and analyzed by ELISA to determine their human AFP levels. Human AFP ELISA kits, mouse interleukin-6 (IL6) ELISA kit, and mouse tumor necrosis factor-α (TNFα) ELISA kit were purchased from Abcam. ELISA was performed according to the manufacturer’s instructions. The serum AFP, IL6, and TNFα levels were calculated according to each standard.

### Hematoxylin and eosin staining

The harvested mouse livers were fixed with 4% PFA, embedded in paraffin, and sectioned at 5 μm. The sections were stained with hematoxylin (FUJIFILM Wako) and eosin (HE) Y (FUJIFILM Wako).

### Cell viability tests

Cell viability was assessed by using a WST-8 assay kit (Dojindo) according to the manufacturer’s instructions. Cell viability was also examined by crystal violet staining.

### Cell sorting

Single-cell suspensions of the HepG2 cells were prepared, and then cell sorting was performed using a FACS Aria II (BD Biosciences). Analysis was performed on an FlowJo software (FlowJo LLC, http://www.flowjo.com/).

### Immunohistochemistry

For detection of human AFP, xenograft tumors were harvested. Harvested xenograft tumors were frozen in Tissue-Tek O.C.T. Compound (Sakura Finetek), then sectioned at 10 μm and fixed with 4% paraformaldehyde for 15 min. After incubation with 0.1% Tween 20 and blocking with ImmunoBlock (DS Pharma Biomedical), the sections were incubated with anti-human AFP antibody (Abcam) at 4 °C overnight, followed by incubation with a secondary antibody that had been labeled with Alexa Fluor 488 (Thermo Fisher Scientific) at 4 °C for 1 h.

### Western blotting

The cells were homogenized with RIPA buffer (Thermo Fisher Scientific) containing a protease inhibitor mixture (Sigma-Aldrich). After being frozen and thawed, the homogenates were centrifuged at 15,000 × *g* at 4 °C for 10 min, and the supernatants were collected. The lysates were subjected to SDS–PAGE on 7.5% polyacrylamide gel, and then transferred onto polyvinylidene fluoride membranes (Millipore). After the reaction was blocked with 1% skim milk in TBS containing 0.1% Tween 20 at room temperature for 1 h, the membranes were incubated with mouse anti-Adenovirus-5 E1A (sc-58658, Santa Cruz Biotechnology) or mouse anti-β-Actin antibody (A5316, Sigma-Aldrich) at 4 °C overnight, followed by reaction with anti-mouse IgG, HRP-linked antibodies (Cell Signaling Technology) at room temperature for 1 h. The band was visualized by Chemi-Lumi One Super (Nakalai Tesque) and the signals were read using an LAS-4000 imaging system (FUJIFILM).

### Determination of Ad genome copy numbers

Total DNA, including Ad genomic DNA, was isolated from the transduced cells by using a DNeasy Blood and Tissue Kit (Qiagen). After the isolation, the Ad genome copy numbers were quantified by using StepOnePlus, as we previously described^8,9^.

### Real-time RT-PCR

Total RNA was isolated from cells using ISOGENE (NIPPON GENE). cDNA was synthesized using 500 ng of total RNA with a Superscript VILO cDNA synthesis kit (Thermo Fisher Scientific). Real-time RT-PCR was performed with SYBR Green PCR Master Mix (Applied Biosystems) using a StepOnePlus real-time PCR system (Applied Biosystems). The relative quantitation of target mRNA levels was performed by using the 2^−ΔΔCT^ method. The values were normalized by those of the housekeeping gene, glyceraldehyde 3-phosphate dehydrogenase (*GAPDH*).

## Results

**Fig. 1:**
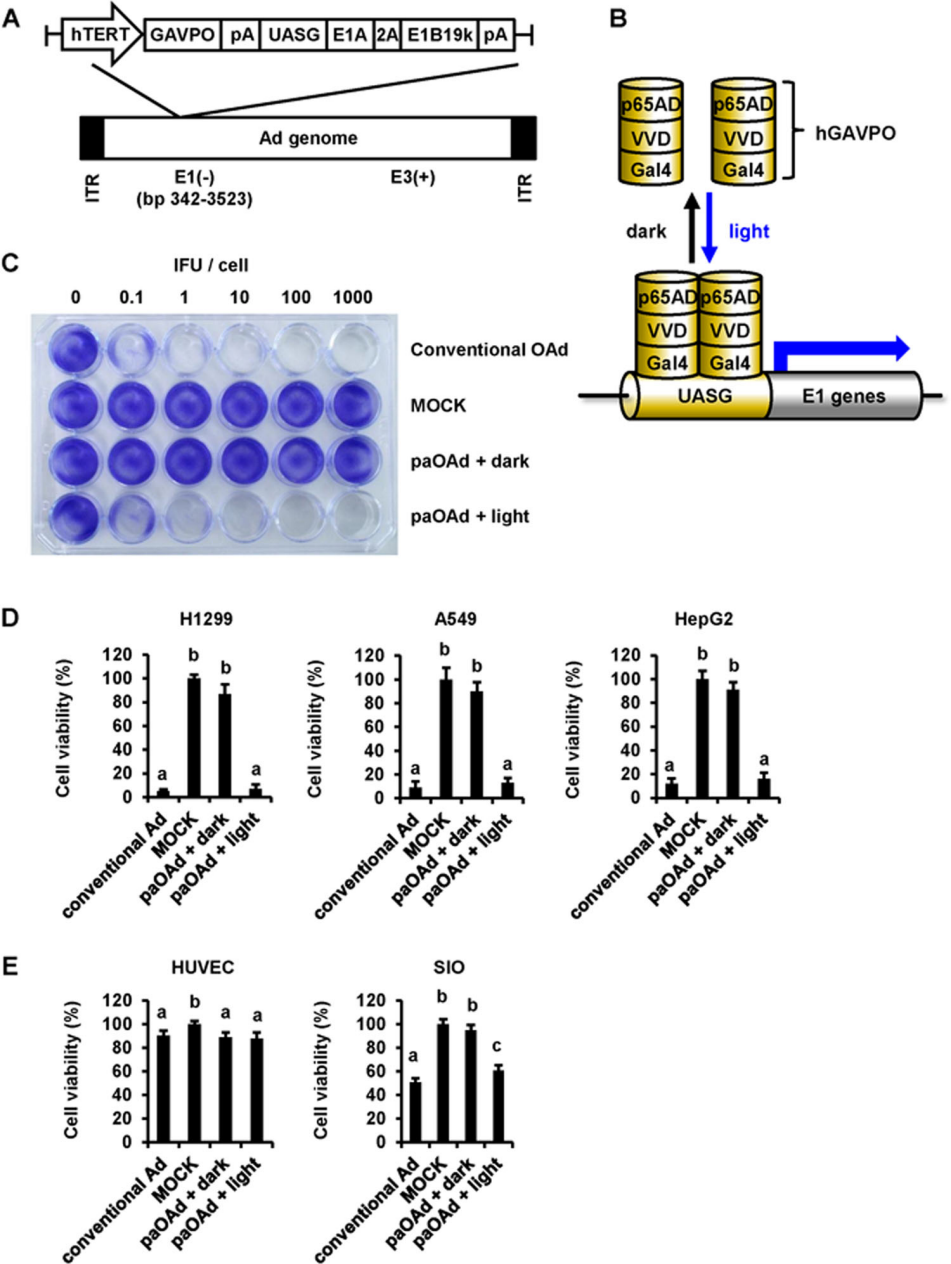
**Design and characterization of the photoactivatable oncolytic adenovirus.** **a** Construction of the photoactivatable oncolytic adenovirus (paOAd). The E1A and E1B19 kDa (E1B19k) genes, which are necessary for adenoviral replication, are inserted downstream of the upstream activating sequence of Gal (UASG). The GAVPO construct consists of sequences encoding VVD (the smallest LOV domain-containing protein), Gal4 (Gal4 residues 1–65), and the p65 activation domain. GAVPO is under the control of the hTERT promoter. The phTERT-GAVPO-pA-UAS-E1A-2A-E1B19k-pA sequence was inserted into the E1 region-deleted human adenovirus type 5 (Ad5) backbone. **b** Schematic overview of the paOAd. Blue light irradiation induces homodimerization of GAVPO, which enables Gal4(65) to bind UASG, thereby inducing the expression of E1A and E1B19k. **c** Crystal violet analysis of the cytopathic effects of paOAd in H1299 tumor cells. The H1299 cells were infected with conventional OAd or paOAd at the indicated infectious units (IFU)/cell for 2 h followed by 0.1 mW/cm^2^ blue light irradiation for 5 days. Five days after infection, the cells were stained with crystal violet. Data are representative of three independent experiments. **d** H1299, A549, and HepG2 cancer cell lines were infected with the paOAd at 10 IFU/cell for 2 h followed by 0.1 mW/cm^2^ blue light irradiation for 5 days. Five days after infection, the cell viability was determined by WST-8 assay. **e** The normal human cells, HUVEC (TERT negative), and human SIO (TERT positive) were infected with the paOAd at 10 IFU/cell for 2 h followed by 0.1 mW/cm^2^ blue light irradiation for 5 days. Five days after infection, the cell viability was determined by WST-8 assay. The data were normalized by the data of the mock-infected group. The results are shown as the mean ± S.E. (*n* = 3). Statistical significance was evaluated by one-way ANOVA followed by Tukey’s post hoc tests. Groups that do not share the same letter had cell viabilities significantly different from each other (*p* < 0.05).

To enhance the safety of TRADs, we attempted to generate a paOAd in this study. We adopted a light switchable transcription factor, GAVPO system^10^ to confer photoactivity to oncolytic adenoviruses (Fig. 1a). The E1A and E1B19 kDa (E1B19k) genes, which are necessary for adenoviral replication, were inserted into the region downstream of the upstream activator sequence of Gal4 (UASG). Blue light irradiation induces homodimerization of GAVPO, which enables Gal4(65) to bind UASG, thereby inducing the expression of E1A and E1B19k (Fig. 1b). We confirmed that the E1A and E1B expression levels in the tumor cells were significantly enhanced by the blue light irradiation (Supplementary Fig. 1). In our paOAd, the GAVPO is under the control of the hTERT promoter. It is expected that the adenoviral genome of paOAd will be replicated in the hTERT-positive cells that are irradiated with blue light. As expected, the copy number of the adenoviral genome in the tumor cells was increased by the blue light irradiation (Supplementary Fig. 2). To examine the cytopathic effects of paOAd in the tumor cells (H1299), a crystal violet analysis was performed (Fig. 1c). The cytopathic effects of paOAd were confirmed when the cells were irradiated with blue light, but not under the dark condition. This result indicates that paOAd can achieve its cytopathic effects in a blue light irradiation-dependent manner.

To examine the on-target effects of paOAd, various cancer cell lines were infected with the paOAd followed by blue light irradiation (Fig. 1d). The cell viabilities of H1299, A549, and HepG2 cells were significantly decreased by paOAd infection followed by blue light irradiation. Importantly, there was little change in the cell viabilities of paOAd-infected cells that were cultured under the dark condition. These results suggest that paOAd can achieve its cytopathic effects in cancer cells in a blue light irradiation-dependent manner. To examine the off-tumor effects of paOAd, normal cells were infected with paOAd followed by blue light irradiation (Fig. 1e). For this experiment, we used HUVEC (hTERT-negative cells) and human SIO (hTERT-positive cells). As expected, the cell viability was largely decreased in the human SIO by conventional OAd infection. However, the cell viabilities in both HUVEC and human SIO were not changed by paOAd infection unless the cells were irradiated with blue light. These results suggest that paOAd has lower off-tumor toxicity than conventional OAd.

**Fig. 2:**
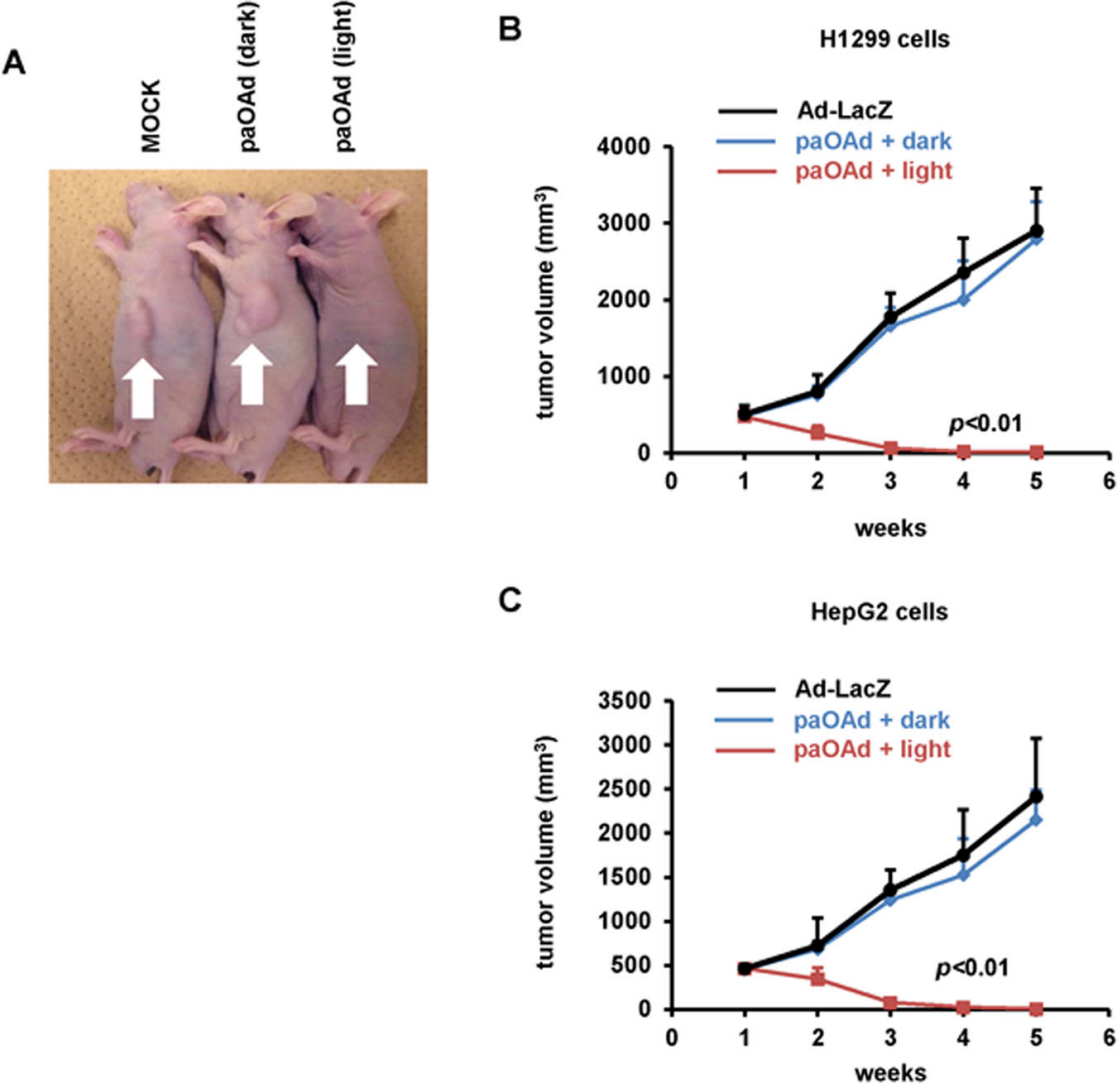
**In vivo antitumor effects of paOAd in a subcutaneous xenograft model mice**. BALB/c *nu/nu* mice bearing H1299 or HepG2 subcutaneous xenograft tumors were intratumorally administered 10^7^ IFU of paOAd at days 0 and 3. H1299 xenograft tumors were irradiated with 90 mW/cm^2^ blue light from day 0 to day 10 (8 h/day). **a** The macroscopic appearance of H1299 tumors in *nu/nu* mice at 5 weeks after treatment. **b**, **c** Growth of H1299 (**b**) or HepG2 (**c**) xenograft tumors was expressed as the mean tumor volume ± S.E. Data were generated from 16 mice for each group. Replication-deficient LacZ-expressing Ad (Ad-LacZ) was used as a control. Statistical significance was evaluated by two-way repeated ANOVA followed by Tukey’s post hoc tests. At 2, 3, 4, and 5 weeks after the illumination, the mean tumor volumes in the “paOAd + light” group were higher than those of the other groups (*p* < 0.01).

To examine the in vivo antitumor effects of paOAd, a subcutaneous xenograft model was used. Subcutaneous xenograft tumors in nu/nu mice were intratumorally administered the paOAd followed by blue light irradiation (paOAd + light group). The subcutaneous xenograft tumors almost entirely disappeared following this treatment (Fig. 2a). At 2, 3, 4, and 5 weeks after the illumination, the mean tumor volumes of the paOAd + light group were lower than those of the other groups (Fig. 2b, c). These results suggest that the paOAd exhibited in vivo antitumor effects against the subcutaneous xenograft model.

**Fig. 3:**
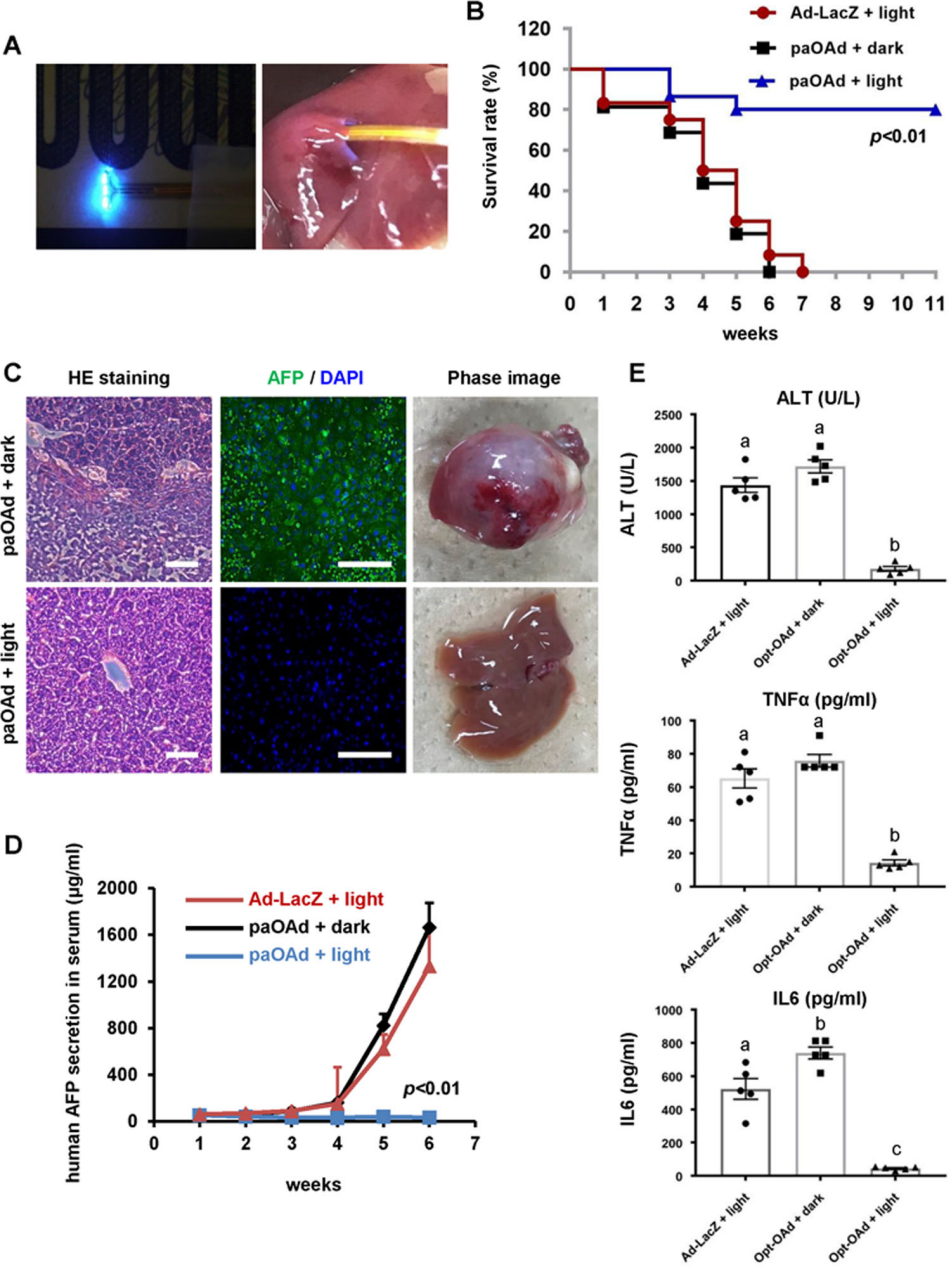
**In vivo antitumor effects of paOAd in a liver cancer model.** *Rag2-Il2rg* double-knockout mice bearing HepG2 liver xenograft tumors were intravenously administered 5 × 10^9^ IFU of paOAd at days 0 and 3. HepG2 xenograft tumors were irradiated with 1 mW/cm^2^ blue light using an implantable LED device from day 0 to day 14 (6 h/day). **a** The liver was irradiated with the implantable blue LED device. **b** The survival rate of the mice after infection of paOAd followed by blue light irradiation was examined. Statistical significance was evaluated by the log-rank test. The survival rate of “paOAd + light” group was higher than those of other groups (*p* < 0.01). **c** The phase images and HE staining images of the mouse liver are shown. The AFP (green) expression was examined by immunohistochemical analysis. Nuclei were counterstained with DAPI (blue). **d** The human AFP secretion in the mouse serum after infection of paOAd followed by blue light irradiation was examined. The results are shown as the mean ± S.E. Statistical significance was evaluated by two-way repeated ANOVA followed by Tukey’s post hoc tests. At 5 and 6 weeks after the illumination, the human AFP secretion in the “paOAd + light” group was lower than those of the other groups (*p* < 0.01). **e** The ALT value, TNFα secretion, and IL6 secretion in the mouse serum after infection of paOAd followed by blue light irradiation were examined. The results are shown as the mean ± S.E. Statistical significance was evaluated by one-way ANOVA followed by Tukey’s post hoc tests. Groups that do not share the same letter are significantly different from each other (*p* < 0.05). Data were generated from 13 mice for each group. Scale bars represent 100 μm.

It is known that blue light does not readily penetrate tissue. Although, in Fig. 2, blue light was successfully transmitted into the subcutaneous space, it is impossible to transmit blue light into the deep tissue regions. Recently, LED chips of <300 µm × 300 µm size have been made commercially available from various manufacturers. Such small blue LEDs could be attached to a catheter, allowing the illumination of deep tissues, such as the prostate and liver. Therefore, we next used a liver cancer model to examine the antitumor effects of paOAd in deep tissues, such as the liver. *Rag2-Il2rg* double-knockout mice with liver cancer were intravenously administered paOAd followed by blue light irradiation using an implantable blue LED device (Fig. 3a). The survival rate of the mice after paOAd infection was significantly increased by blue light irradiation (Fig. 3b). At week 6 after the initial paOAd infection, HE staining images showed that the HepG2 cells had almost entirely disappeared from the mouse liver (Fig. 3c). At week 6 after the initial paOAd infection, the human AFP (a specific liver cancer marker)-positive cells were almost disappeared (Fig. 3c). Also, the human AFP (a specific liver cancer marker) secretion in the mouse serum after paOAd infection was significantly decreased by blue light irradiation (Fig. 3d). Furthermore, ALT value (alanine aminotransferase, biochemical markers of hepatocellular injury), TNFα (inflammatory cytokine involved in liver inflammation) secretion, and IL6 (inflammatory cytokine involved in liver inflammation) secretion in the mouse serum after paOAd infection were significantly decreased by blue light irradiation (Fig. 3e). We also confirmed that paOAd transferred to the spleen and intestinal tract less than conventional OAd (Supplementary Fig. 3). In addition, the E1A expression levels in nonirradiated organs of mice administered with paOAd were less than those of mice administered with conventional OAd (Supplementary Fig. 4). On the other hand, the amount of Ad genome in the liver of mice administered with conventional OAd was similar to that of mice administered with paOAd. For unknown reasons, it is assumed that light irradiation made it difficult for the Ad to be released from the liver. These results suggest that the paOAd exhibited in vivo antitumor effects in the liver cancer model.

**Fig. 4:**
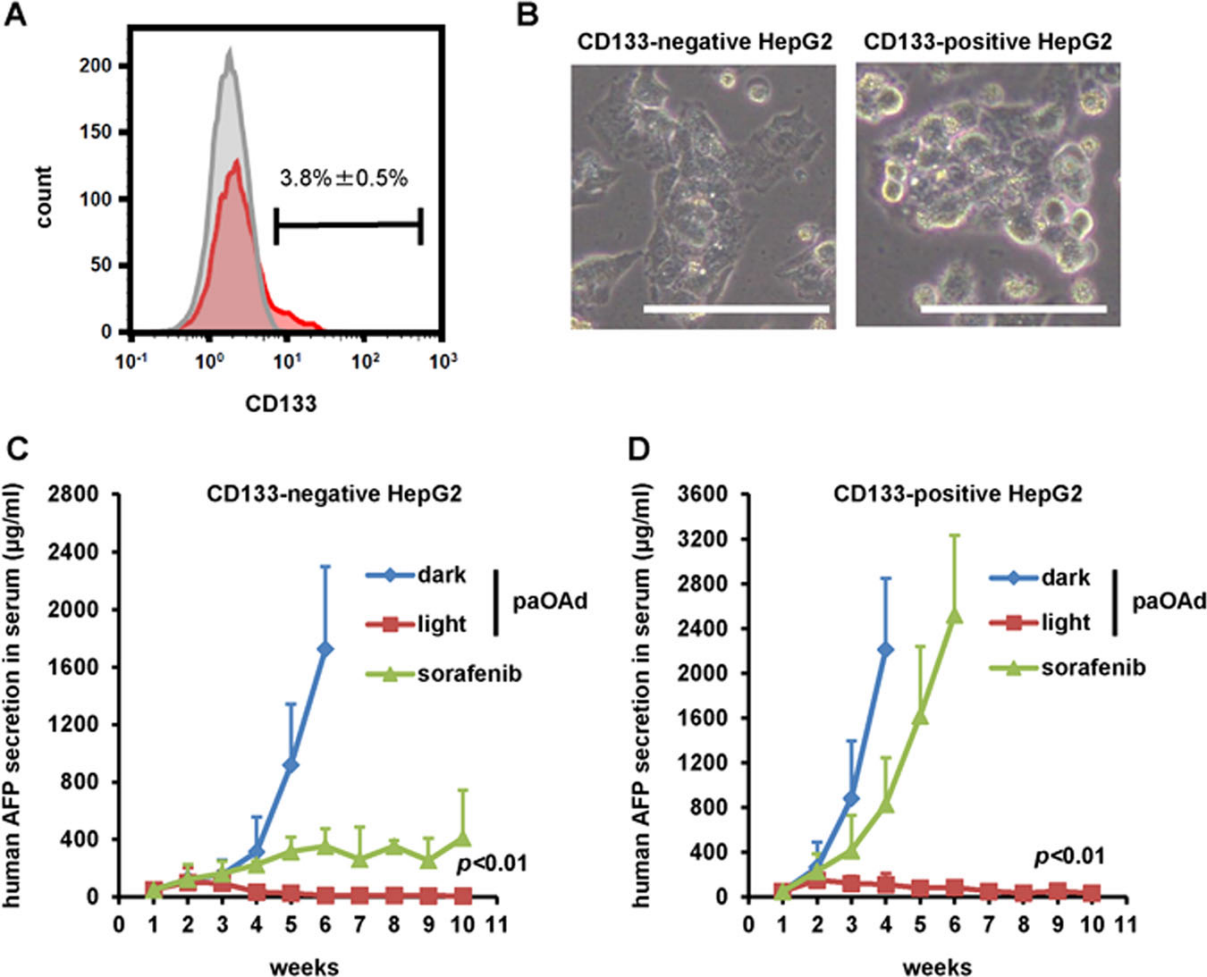
**In vivo antitumor effects of paOAd in a xenograft model mice with cancer stem cells.** CD133-positive HepG2 cells were used as a model of cancer stem cells. **a** The CD133-positive cells were collected using a cell sorter. **b** The phase images of CD133-negative and -positive HepG2 cells are shown. **c** In the *Rag2-Il2rg* double-knockout mice transplanted with CD133-negative HepG2 cells, the AFP secretion in the mouse serum after paOAd infection was measured by ELISA. The *Rag2-Il2rg* double-knockout mice transplanted with CD133-negative HepG2 cells were treated orally for 10 weeks with sorafenib at 30 mg/kg. The results are shown as the mean ± S.E. Statistical significance was evaluated by two-way repeated ANOVA followed by Tukey’s post hoc tests. At 5 and 6 weeks after the illumination, the human AFP secretion in the “paOAd + dark” group was higher than those of the other groups (*p* < 0.01). **d** In the *Rag2-Il2rg* double-knockout mice transplanted with CD133-positive HepG2 cells, the AFP secretion in the mouse serum after paOAd infection was measured by ELISA. The *Rag2-Il2rg* double-knockout mice transplanted with CD133-positive HepG2 cells were treated orally for 10 weeks with sorafenib at 30 mg/kg. The results are shown as the mean ± S.E. (*n* = 3). Statistical significance was evaluated by two-way repeated ANOVA followed by Tukey’s post hoc tests. At 4, 5, and 6 weeks after the illumination, the human AFP secretion in the “paOAd + light” group was lower than those of the other groups (*p* < 0.01). Data were generated from 11 mice for each group. Scale bars represent 50 μm.

Current cancer therapies, including chemotherapy and radiation therapy, do not show sufficient therapeutic effects on cancer stem cells^11^. On the other hand, it is reported that oncolytic virus can kill cancer stem cells^12,13^. In this study, CD133-positive HepG2 cells were used as a model of cancer stem cells (Fig. 4a, b). In the *Rag2-Il2rg* double-knockout mice transplanted with CD133-negative HepG2 cells, the AFP secretion in the mouse serum after paOAd infection was significantly decreased by blue light irradiation (Fig. 4c). In addition, Sorafenib, which is widely used in the treatment of liver cancer, also showed a therapeutic effect. The AFP secretion in the mouse serum after paOAd infection was significantly decreased by blue light irradiation in the *Rag2-Il2rg* double-knockout mice transplanted with CD133-positive HepG2 cells, while that did not change by Sorafenib treatment (Fig. 4d). These results indicate that paOAd can exert therapeutic effects on cancer stem cells.

## Discussion

Here, we succeeded in generating a paOAd with high antitumor potency but a low level of off-tumor effects. This paOAd should enable the performance of adenovirus-based oncolytic cancer therapy with much higher tumor selectivity than ever before. However, some issues must be addressed before advancing the paOAd to clinical trials. First, the method for blue light irradiation has to be improved. A less invasive blue light irradiation method would be preferable for clinical trials. Although we used a blue LED device with a power cord, it would be better to use a wireless blue LED device with an autonomous power supply. Because it is known that blue light does not readily penetrate tissue, we have to use long-wavelength light in the next project. By using upconverting nanoparticles^14^, infrared light, which can penetrate tissue, can be converted into blue light in vivo.

Second, the GAVPO system has to be improved. In our in vivo experiment, mice were irradiated with blue light for 6–8 h/day. Long-term blue light irradiation might cause phototoxicity and unexpected heat generation. Recently, various activation domains (such as SAM, VPR, and SunTag)^15^, that have higher activity than the p65 activation domain (p65AD), have been developed. By using these domains, it might be possible to induce higher E1 gene expression. In addition, it might be better to use a photoreceptor, which has higher light sensitivity than Vivid, such as Magnet system^16^.

Third, the antitumor effects would be enhanced by arming it with therapeutic genes^17,18^. The antitumor effects of oncolytic viruses have previously been improved by arming them with the genes encoding IL-12, CD40 ligand, and granulocyte macrophage colony-stimulating factor. By adding modifications to adenovirus to avoid innate immunity or optimizing the administration method, the oncolytic potency of paOAd may be increased. Light-activated CRISPR/Cas9 effector (LACE) or Split-CPST2.0 systems enable the induction of endogenous gene expressions by using blue light irradiation^9,19,20^. Therefore, it would be possible to regulate adenoviral replication and therapeutic gene expression by introducing a LACE or Split-CPTS2.0 system into paOAd. In short, although there are many issues to be resolved before the medical application of paOAd, paOAd has clear potential as a safe and therapeutically effective cancer therapy.

## Supplementary information

Supplementary Information

Supplementary Information

Supplementary Information

Supplementary Information

Supplementary Information
