# Supplementary material for: Photoactivatable oncolytic adenovirus for optogenetic cancer therapy
Source: Cell Death Dis. 2020 Jul 23;11(7):570. doi: 10.1038/s41419-020-02782-6 (PMC7378209; doi:10.1038/s41419-020-02782-6)
Supplement: Supplementary file 1 — Supplementary Information [file 41419_2020_2782_MOESM1_ESM.docx]

**Supplemental figure legends**

**Figure S1 Expression analysis of adenoviral E1A and E1B genes**

The H1299 cells and HUVEC were infected with paOAd at 10 IFU/cell for 2 hr followed by 0.1 mW/cm^2^ blue light irradiation for 1 day. One day after infection, the cells were collected and the expression analysis of E1A and E1B was performed. (**A, B**) The *E1A* and *E1B* expression levels in H1299 cells (**A**) and HUVEC (**B**) were examined by real-time RT-PCR. The gene expression levels in non-irradiated cells were taken as 1.0. (**C**) The E1A expression levels were examined by western blotting. The results are shown as the mean ± S.E (*n*=3).

**Figure S2 Blue light-dependent replication ability of paOAd**

The A549 cells were infected with conventional OAd or paOAd at 10 IFU/cell for 2 hr followed by 0.1 mW/cm^2^ blue light irradiation for 12, 24 and 48 hours. The copy number of adenoviral genome was examined. The results are shown as the mean ± S.E (*n*=3).

**Figure S3 Quantification of adenoviral genome in the various mouse organs treated with OAd or paOAd**

*Rag2-Il2rg* double-knockout mice bearing HepG2 liver xenograft tumors were intravenously administered 5×10^9^ IFU of paOAd at day 0 and 3. HepG2 xenograft tumors were irradiated with 1 mW/cm^2^ blue light using an implantable LED device from day 0 to day 14 (6 hr/day). As a control, *Rag2-Il2rg* double-knockout mice bearing HepG2 liver xenograft tumors were intravenously administered 5×10^9^ IFU of conventional OAd at days 0. At day 21, the copy number of adenoviral genome in various mouse organs was examined. The results are shown as the mean ± S.E (*n*=8).

**Figure S4 Expression analysis of adenoviral E1A gene in the various mouse organs treated with OAd or paOAd**

*Rag2-Il2rg* double-knockout mice bearing HepG2 liver xenograft tumors were intravenously administered 5×10^9^ IFU of paOAd at day 0 and 3. HepG2 xenograft tumors were irradiated with 1 mW/cm^2^ blue light using an implantable LED device from day 0 to day 7 (6 hr/day). As a control, *Rag2-Il2rg* double-knockout mice bearing HepG2 liver xenograft tumors were intravenously administered 5×10^9^ IFU of conventional OAd at days 0 and 3. At day 7, the E1A expression levels in various mouse organs were examined. The gene expression levels of E1A in bone marrow of OAd-infected mice were taken as 100. The results are shown as the mean ± S.E (*n*=8).
