# Supplementary figures and images for: Photoactivatable oncolytic adenovirus for optogenetic cancer therapy
Source: Cell Death Dis. 2020 Jul 23;11(7):570. doi: 10.1038/s41419-020-02782-6 (PMC7378209; doi:10.1038/s41419-020-02782-6)

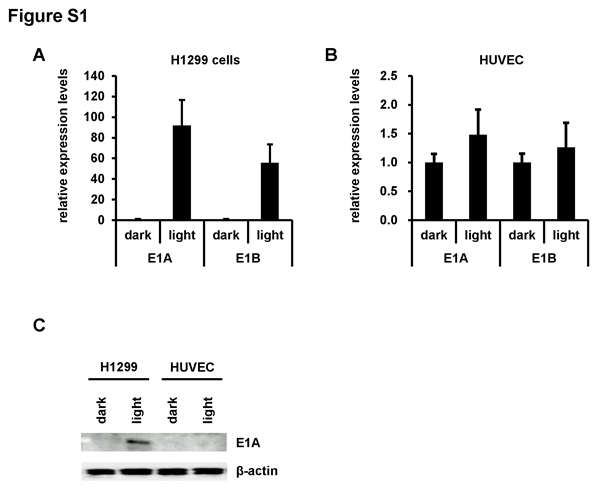

Supplement: Supplementary file 2 — Supplementary Information [file 41419_2020_2782_MOESM2_ESM.tif]

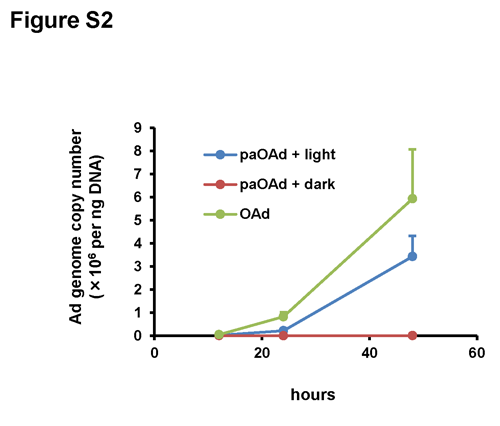

Supplement: Supplementary file 3 — Supplementary Information [file 41419_2020_2782_MOESM3_ESM.tif]

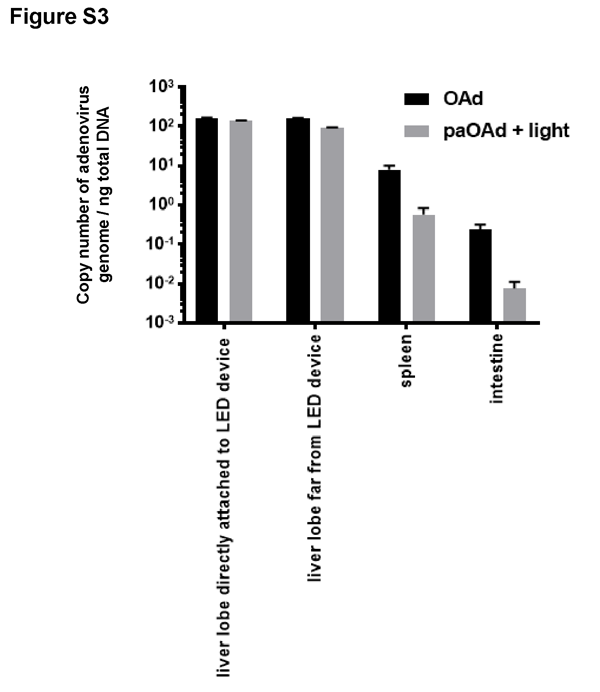

Supplement: Supplementary file 4 — Supplementary Information [file 41419_2020_2782_MOESM4_ESM.tif]

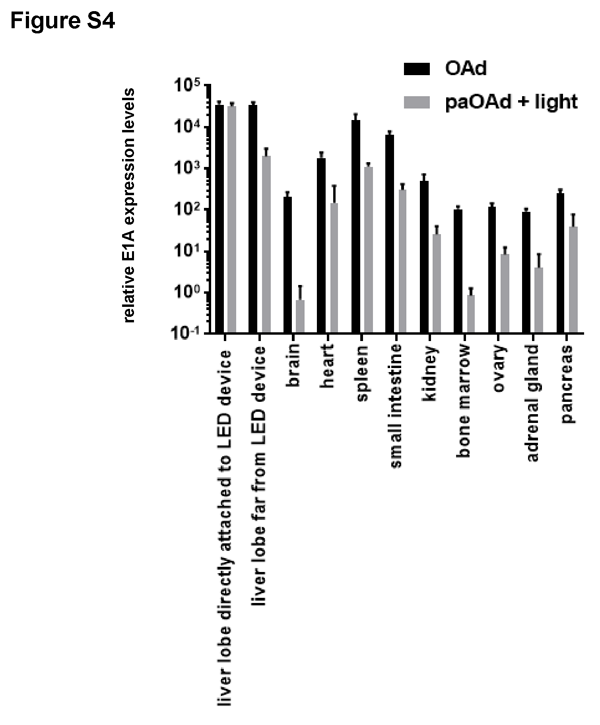

Supplement: Supplementary file 5 — Supplementary Information [file 41419_2020_2782_MOESM5_ESM.tif]
